# An RNA Motif That Enables Optozyme Control and Light‐Dependent Gene Expression in Bacteria and Mammalian Cells

**DOI:** 10.1002/advs.202304519

**Published:** 2024-01-16

**Authors:** Georg Pietruschka, Américo T. Ranzani, Anna Weber, Tejal Patwari, Sebastian Pilsl, Christian Renzl, David M. Otte, Daniel Pyka, Andreas Möglich, Günter Mayer

**Affiliations:** ^1^ Life and Medical Sciences (LIMES) University of Bonn Gerhard‐Domagk‐Str.1 53121 Bonn Germany; ^2^ Lehrstuhl für Biochemie, Photobiochemie University of Bayreuth Universitätsstraße 30 95440 Bayreuth Germany; ^3^ Center of Aptamer Research & Development University of Bonn Gerhard‐Domagk‐Str. 1 53121 Bonn Germany; ^4^ Present address: Bond Life Sciences Center University of Missouri Columbia MO 65211 USA

**Keywords:** aptamers, LOV‐domains, optogenetics, ribozymes, SELEX, synthetic biology

## Abstract

The regulation of gene expression by light enables the versatile, spatiotemporal manipulation of biological function in bacterial and mammalian cells. Optoribogenetics extends this principle by molecular RNA devices acting on the RNA level whose functions are controlled by the photoinduced interaction of a light‐oxygen‐voltage photoreceptor with cognate RNA aptamers. Here light‐responsive ribozymes, denoted optozymes, which undergo light‐dependent self‐cleavage and thereby control gene expression are described. This approach transcends existing aptamer‐ribozyme chimera strategies that predominantly rely on aptamers binding to small molecules. The optozyme method thus stands to enable the graded, non‐invasive, and spatiotemporally resolved control of gene expression. Optozymes are found efficient in bacteria and mammalian cells and usher in hitherto inaccessible optoribogenetic modalities with broad applicability in synthetic and systems biology.

## Graphical abstract

Introducing optozymes for controlling gene expression with light. By harnessing molecular RNA devices, optozymes allow accurate manipulation of biological function in both bacterial and mammalian cells. Diverging from traditional strategies, optozymes rely on the photoinduced interaction of a light‐oxygen‐voltage photoreceptor with RNA aptamers, enabling non‐invasive and spatiotemporally resolved control. Optozymes pave the way for wide applications in regulating gene expression in synthetic and systems biology.

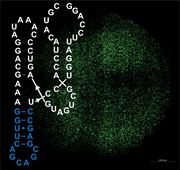

## 1 Introduction

RNA elements that respond to specific trigger signals are valuable tools in synthetic biology, biotechnology, and biomedical applications as they allow for the control and analysis of gene expression.^[^ ^1^ ^]^ The underlying interactions between the RNA and trigger signals are achieved by aptamer domains, i.e., RNA structures that bind target molecules with high specificity and affinity. For example, the recently emerged fluorogenic RNAs, such as spinach, broccoli and pepper, enable the construction of light‐up sensors that directly respond to concentration variations of small molecules, other RNAs, and proteins in cells.^[^ ^2^, ^3^, ^4^, ^5^, ^6^, ^7^ ^]^ On the level of transcriptional control, guide RNAs of the CRISPR/Cas system have been modified with aptamer domains, thus enabling the trigger‐dependent recruitment of cognate‐binding proteins for the transactivation of gene expression.^[^ ^8^, ^9^, ^10^ ^]^ Likewise, small regulatory RNA molecules, e.g., siRNAs and micro RNAs have been modified to respond to small molecules, proteins, and light triggers,^[^ ^11^, ^12^, ^13^ ^]^ thereby granting exogenous control of gene expression on the level of mRNA. Moreover, RNA aptamers were embedded into self‐cleaving RNAs, e.g., hammerhead or twister ribozymes, to generate aptazyme variants with signal‐dependent catalytic activity. Insertion of aptazymes into the untranslated regions (UTR) of mRNAs has proven useful in gaining control of mRNA translation and stability.^[^ ^14^, ^15^, ^16^, ^17^, ^18^, ^19^ ^]^ Whereas the above strategies predominantly relied on aptamers binding to small molecules such as theophylline, doxycycline, and aminoglycosides, light‐responsive systems are scarce.

Sensory photoreceptors serve as light‐sensing proteins in different organisms to convert stimuli of specific wavelengths into biological responses.^[^ ^20^, ^21^ ^]^ One class of photoreceptors is represented by the so‐called light‐oxygen‐voltage (LOV) receptors, which use flavin nucleotides, most frequently flavin mononucleotide, as chromophores to obtain light sensitivity.^[^ ^22^ ^]^ Upon irradiation with blue light, a covalent bond forms between the flavin molecule and an adjacent cysteine residue, eventually leading to a structural rearrangement of the α‐helical element that conjoins the LOV sensor domain with an effector domain.^[^ ^23^, ^24^ ^]^ In this way, the effector function, e.g., kinase activity is regulated by light. This process is reversible, and in darkness, the light‐activated receptor thermally reverts to the non‐irradiated state. LOV domains have been used to engineer light‐regulated effectors for optogenetics, thereby achieving spatiotemporal control of cellular physiology, e.g., DNA binding or cell migration.^[^ ^25^ ^]^ The recently identified photoreceptor *Nm*PAL has a unique domain architecture with a C‐terminal LOV domain regulating access to a preceding ANTAR domain. Once activated by blue light, *Nm*PAL binds to short artificial RNA hairpins,^[^ ^26^ ^]^ thus enabling optoribogenetics, i.e., the regulation of cellular RNA‐dependent processes by light, e.g., transcription, translation, and RNA interference.^[^ ^9^, ^11^, ^26^, ^27^ ^]^

Using SELEX, an in vitro selection method to isolate short nucleic acid molecules that bind to targets of interest from combinatorial libraries,^[^ ^28^, ^29^ ^]^ we initially identified two RNA hairpins, termed motif 1 and motif 2 (Figure S2a, Supporting Information), which bind *Nm*PAL in a light‐dependent manner.^[^ ^26^ ^]^ Although both hairpins share structural and sequence motifs, their suitability for cellular applications differs significantly. Motif 1 was found to be more effective for prokaryotic applications and less active in the cellular environments of mammalian cells. By contrast, motif 2 was more suitable for applications in mammalian cells.

Here, we describe the identification of an additional RNA hairpin, dubbed motif 3, by genome mining and SELEX that binds to *Nm*PAL with high affinity and, most importantly, is applies to both prokaryotic and mammalian cells as a versatile synthetic biology tool. We applied motif 3 to create aptazyme variants, termed optozymes in the following, that allow the control of ribozyme self‐cleavage activity by light. Embedding these optozymes into mRNAs enables the efficient light‐dependent regulation of gene expression in mammalian cells and bacteria.

## 2 Results

### 2.1 Genome Mining and SELEX Identifies a New PAL‐Binding Aptamer Motif 3

**Figure 1:**
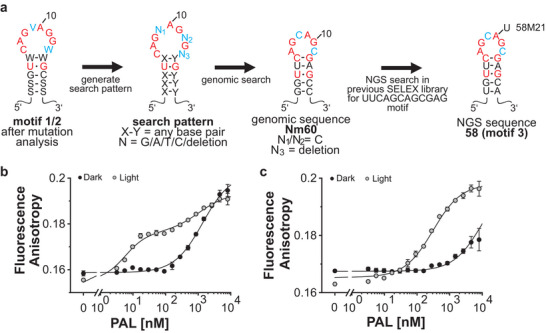
a) Consensus sequence and predicted secondary structure for motifs 1 and 2 based on previous mutational analyses (S = G/C, W = A/U, V = A/C/G).^[^ ^26^ ^]^ Search pattern for the genomic search based on the consensus sequences and predicted secondary structure for motifs 1 and 2. Most promising genomic sequence (Nm60) after validation of the resulting sequences for possible hairpin formation and localization in the 5′‐UTR. Sequence and predicted secondary structure of aptamer 58 identified via next‐generation sequencing analysis of the previously reported aptamer enrichment using *Nm*PAL.^[^ ^26^ ^]^ Fluorescence anisotropy measurements of the interaction of the 18‐nt 58 hairpin (b) and its corresponding mutated version 58.M21 (c) with *Nm*PAL in darkness or under blue light (*λ* = 460 nm). Data in panels (b,c) represent the mean ± s.d. of two replicates. These experiments were repeated at least three times with similar outcome.

*Nm*PAL is a photoreceptor found in *Nakamurella multipartita*, comprising an ANTAR domain flanked by an N‐terminal PAS and a C‐terminal LOV domain.^[^ ^26^ ^]^ ANTAR domains are RNA‐binding proteins functioning as antiterminators during bacterial transcription.^[^ ^30^ ^]^ The natural RNA bound by *Nm*PAL is yet unknown. Based on mutational and binding studies of the previously discovered motifs 1 and 2, we defined a minimal search pattern with which we mined the genome of *N. multipartita* (**Figure** **1**a).^[^ ^31^ ^]^ This search resulted in a sequence candidate, termed Nm60, located in the 5′‐UTR of a “putative PAS/PAC sensor protein” (Gene Bank: ACV78465.1), thus indicating a potential role in the regulation of the downstream gene (Figure S1, Supporting Information). Compared to motif 2, Nm60 has a deletion of nucleotide N3 at the 5′ end of its unpaired loop, thereby resulting in a smaller loop with six nucleotides and an additional C‐G base pair in the predicted stem (Figure 1a). Revisiting the sequencing data of the initial SELEX experiment,^[^ ^26^ ^]^ we further identified a sequence variant, designated 58, that shares the hairpin and stem motif of Nm60.

After 15 SELEX cycles, the frequency of sequence 58 in the enriched library is 20% with an enrichment profile comparable to aptamer 53, a motif 2 bearing sequence (Figure S2, Supporting Information). Indeed, 58 and 53 share the loop nucleotides (AGCAGC) which led us to initially neglect 58 in our previous experiments and consider it a variant of 53. Using fluorescence anisotropy, an affinity of 3.5 nm ± 1.0 nm of 58 binding to light‐adapted *Nm*PAL (*Nm*PAL_L_) was observed, which is 4–5 times stronger compared to the aptamers 04 (motif 1) and 53 (motif 2).^[^ ^26^ ^]^ The affinity to *Nm*PAL's dark‐adapted conformation (*Nm*PAL_D_) was >3 µm, indicating strong discriminatory properties between dark and light conditions (Figure 1b). Notably, the binding isotherm of *Nm*PAL_L_ exhibited a second transition with micromolar affinity corresponding to that observed in darkness. In analogy to non‐binding mutations known for aptamer 53,^[^ ^26^ ^]^ we generated a variant of 58, termed 58M21, that bears an A to U mutation in the loop region (Figure 1a; Figure S2a, Supporting Information). 58M21 still binds to *Nm*PAL_L_ as determined by fluorescence anisotropy, although at an 83‐fold reduced affinity of ≈250 nm ± 20 nm (Figure 1c). These features show that the binding properties of 58 are distinct from those observed by motifs 1 and 2 and, thus, we refer to sequence 58 as motif 3 (Figure 1; Figure S2a, Supporting Information).

### 2.2 Regulation of Gene Expression in Bacteria by Optozymes

**Figure 2:**
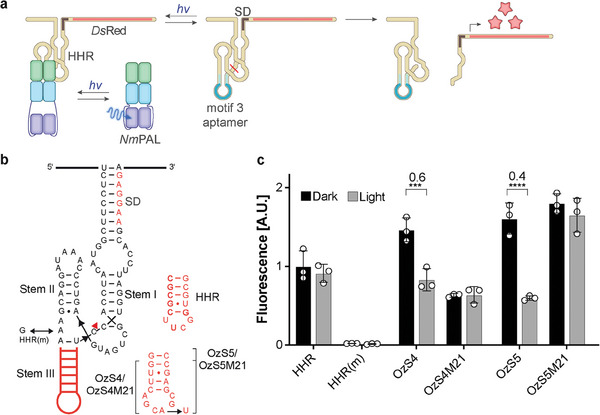
a) Schematic of the optozyme‐controlled expression system in bacterial cells. SD: Shine‐Dalgarno sequence; HHR: Hammerhead ribozyme b) Sequences of the hammerhead ribozyme (HHR), its non‐cleaving mutant (HHR(m)), optozymes OzS5 and OzS4 and their non‐binding M21 mutants. c) Regulation of gene expression by light using the optozymes OzS5 and OzS4 along with the non‐binding controls OzS4M21 and OzS5M21 the mutated HHR(m), and the wild‐type HHR in the presence of *Nm*PAL. The numbers above the bars indicate fold changes calculated from light versus dark conditions. *n* = 3. ^****^: *p* ≤ 0.0001; ^***^: *p* ≤ 0.001, all other light/dark differences were found to be non‐significant.

We next investigated the suitability of motif 3 to regulate gene expression in bacterial and mammalian cells. Aptazymes have previously enabled ligand‐dependent ribozyme cleavage, most likely through an allosteric mechanism.^[^ ^17^ ^]^ We embedded motif 3 in the stem III of the hammerhead ribozyme (HHR) from *Schistosoma mansoni* (Smα) to gain light control over HHR self‐cleavage activity (**Figure** **2**a,b). Our design enables light‐dependent recruitment of *Nm*PAL to the aptazyme and, thus, a potential steric or structural modulation of the aptazyme self‐cleavage activity. Because of the light‐dependency of these aptazymes, we termed them optozymes (Oz). We generated optozyme variants with 5 bp (OzS5) and 4 bp (OzS4) stems that conjoin the motif 3 aptamer with the ribozyme domain (Figure 2b). To assess light control of HHR activity in bacteria, we positioned the optozymes in the 5′‐UTR of the fluorescent reporter gene *Ds*Red such that the Shine Dalgarno (SD) sequence overlaps with stem I of the HHR.^[^ ^17^ ^]^ Thereby, the non‐cleaved optozyme sequesters the SD, whereas optozyme cleavage makes the SD accessible for the bacterial ribosome (Figure 2a). Reporter fluorescence was measured in dependence of the irradiation status. These data indicate that OzS4 and OzS5 left HHR self‐cleavage intact in bacteria and revealed increased *Ds*Red expression levels compared to the HHR (Figure 2c). In the presence of *Nm*PAL both optozyme constructs revealed light‐dependent expression of *Ds*Red, with gene expression lowered under irradiation (Figure 2c), thus indicating that *Nm*PAL binding impaired optozyme self‐cleavage. Notably, OzS5 showed a stronger reduction of gene expression in light compared to OzS4. As controls, neither the motif 3‐point mutants of the optozymes (Figure S2a, Supporting Information), nor the unmodified HHR, nor a cleavage‐deficient mutated version of the HHR (HHR(m)) showed any light‐dependency of gene expression (Figure 2c). Of note, the motif 3‐point mutant of OzS4 (OzS4M21) showed less cleavage activity in darkness compared to OzS4. This feature is not observed in the OzS5 construct and most likely is due to a stronger impact of the mutation on the conformation of the optozyme with the shorter stem.

### 2.3 Regulation of Gene Expression in Mammalian Cells by Optozymes

**Figure 3:**
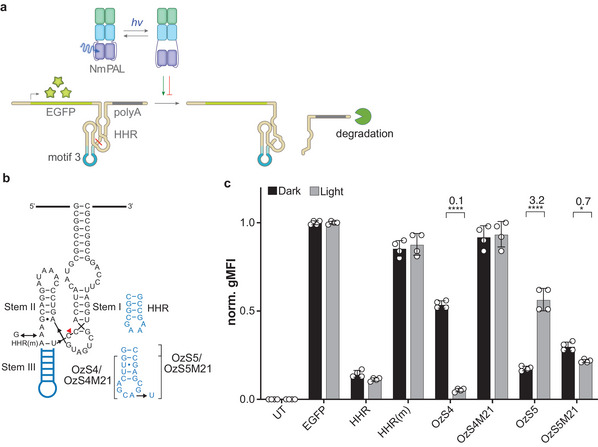
a) Schematic representation of the optozyme‐controlled expression system in mammalian cells. Light‐induced binding of PAL to motif 3 embedded in the stem III of the Hammerhead ribozyme either activates or deactivates the self‐cleavage of the ribozyme and thus gene expression. b) Sequences of the intact hammerhead ribozyme (HHR), its non‐cleaving mutant (HHR(m)), optozymes OzS4 and OzS5 and their non‐binding M21 mutant. c) Regulation of gene expression by light using optozymes OzS4 and OzS5 along with the non‐binding controls OzS4M21 and OzS5M21, the mutated HHRm, and the wild‐type HHR. UT refers to untransfected controls. The numbers above the bars indicate fold changes calculated from light versus dark conditions. norm. gMFI: normalized geometric mean fluorescence intensity; *n* = 2 in duplicates. ^****^: *p* ≤ 0.0001; ^*^: *p* ≤ 0.05, all other light/dark differences were found to be non‐significant.

For gaining light‐control of gene expression in mammalian cells, variants of the above optozymes with adjusted HHR stem I were embedded into the 3′‐untranslated region (UTR) of mammalian mRNAs encoding *EGFP* (**Figure** **3**a,b).^[^ ^32^ ^]^ This positioning has been shown to enable ribozyme‐mediated cleavage of the 3′‐poly‐A tail and to thereby modulate mRNA stability and protein synthesis in mammalian cells.^[^ ^33^, ^34^ ^]^ Using flow cytometry green reporter fluorescence was measured in HEK293T cells co‐transfected with plasmids harboring different HHR variants and a second plasmid encoding either for mCherry or mCherry‐*Nm*PAL (Figure S2e, Supporting Information). In the absence of *Nm*PAL, the integration of motif 3 aptamer impaired HHR activity almost completely for OzS4 and moderately for OzS5, as indicated by high fluorescence readings (Figure 3c). These observations contrast with the experiments in bacteria where the aptamer integration preserved self‐cleavage in both optozymes. When combined with mCherry‐PAL, OzS4 and OzS5 elicited light‐dependent expression of EGFP (Figure 3c). The wild‐type HHR and the incapacitated HHR(m) variant gave rise to low and high fluorescence, respectively, independently of light and the presence of *Nm*PAL. Of note, the optozyme constructs with different stem lengths resulted in opposing light‐dependent behavior. While the optozyme with the 5 bp stem (OzS5) revealed an induction of EGFP expression upon irradiation, the 4 bp stem construct (OzS4) suppressed gene expression under these conditions (Figure 3c; Figure S2c, Supporting Information). This divergent behavior indicates a more pronounced self‐cleavage of OzS5 in darkness than in light, and vice versa in the case of OzS4. The point mutants of motif 3 in the optozyme constructs (OzS4M21 and OzS5M21) abolished light responses, with EGFP expression levels in darkness being low for OzS5M21 but high for OzS4M21 (Figure 3c). This behavior reflects the different intrinsic propensities for self‐cleavage of the optozymes OzS5 and OzS4, but also may indicate a putative interaction of *Nm*PAL with the optozymes in darkness. In line with the reporter‐gene data, in vitro interaction assays using cleavage‐deficient optozymes verify the light‐dependent binding of *Nm*PAL to OzS4 and OzS5 (Figure S2d, Supporting Information), but not to the non‐binding point mutants OzS5M21 and OzS4M21, nor to HHR(m) (Figure S2d, Supporting Information).

**Figure 4:**
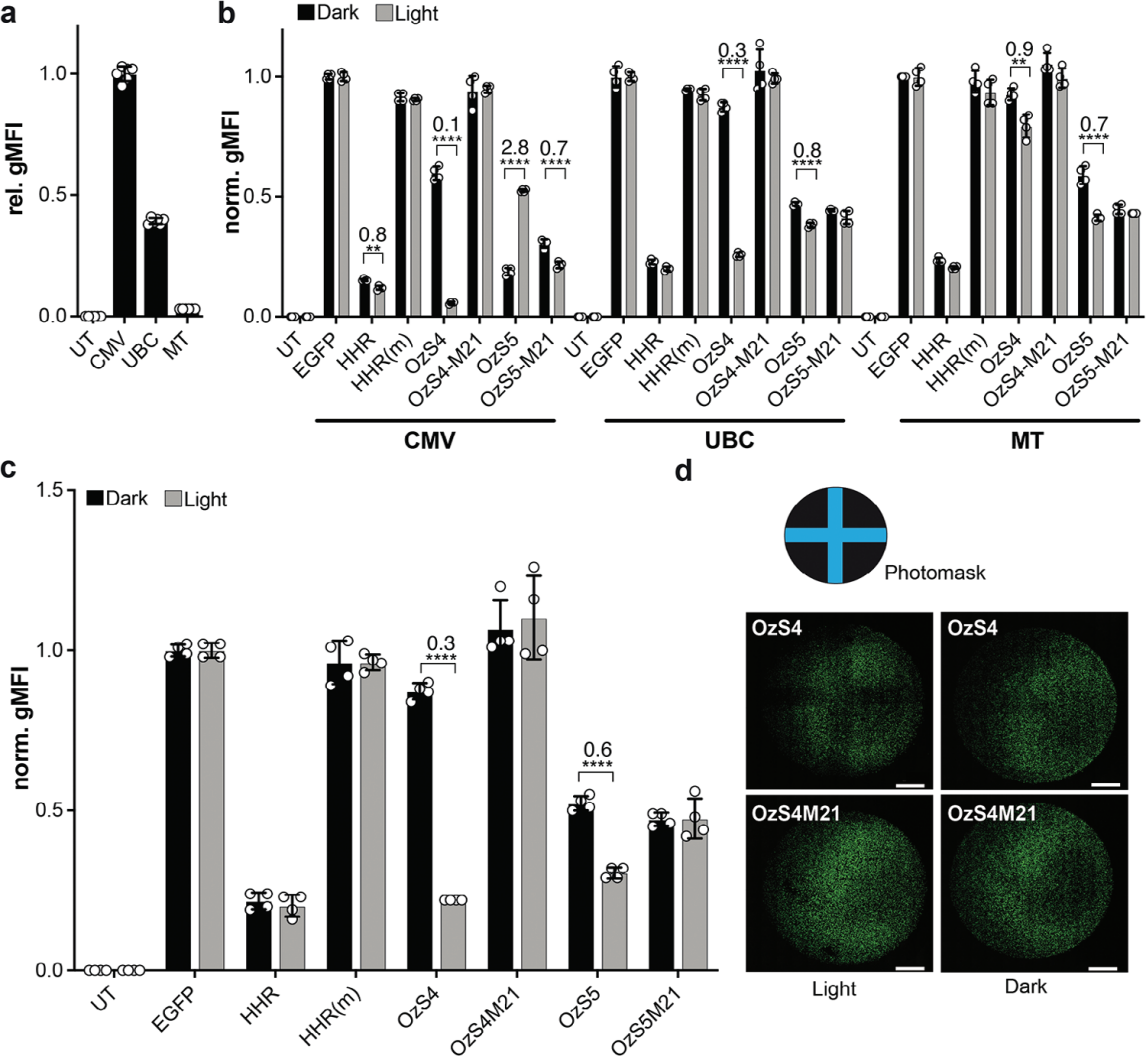
a) Relative expression of mCherry‐*Nm*PAL in HEK cells driven by CMV, Ubc or MT promoters. Data were normalized to those for CMV which was assigned an arbitrary value of 1. UT: Untransfected. b) Light‐dependent expression of EGFP in cells having the optozymes OzS4 or OzS5, or non‐binding mutants (OzS4M21 and OzS5M21) in the 3′‐UTR and mCherry‐*Nm*PAL driven by CMV, Ubc, or MT promoters. HHR: Hammerhead ribozyme; HHR(m): self‐cleavage‐deficient HHR; c) Light‐dependent expression of EGFP in HEK293 cells stably expressing mCherry‐PAL^[^ ^11^ ^]^ and having the optozymes OzS4 or OzS5, or non‐binding mutants (OzS4M21 and OzS5M21) in the 3′‐UTR. *n* = 2, (a) in triplicates, (b,c) in duplicates. ^****^: *p* ≤ 0.0001; ^**^: *p* ≤ 0.01, all other light/dark differences were found to be non‐significant; norm. gMFI: normalized geometric mean fluorescence intensity. The numbers above the bars indicate fold changes calculated from light versus dark conditions. d) Spatial expression of EGFP in HEK293 cells using the optozyme OzS4 or the non‐binding mutant OzS4M21 in the 3′‐UTR. Irradiation was done using the photomask shown. Scale bar: 2000 µm.

The comparison of the reporter‐gene data in the absence and presence of *Nm*PAL provides insight into the origin of the disparate light responses of OzS4 and OzS5 (Figure 3c; Figure S2e, Supporting Information). In darkness, the OzS5 variant exhibited lower reporter fluorescence in the presence of *Nm*PAL than in its absence. Blue light elevated reporter fluorescence in the presence of *Nm*PAL to levels similar to in its absence. These data indicate interactions between OzS5 and the dark‐adapted *Nm*PAL_D_ state, consistent with the micromolar binding to the motif 3 aptamer observed by fluorescence anisotropy (Figure 1b). Because this dark‐state activity might correlate with *Nm*PAL concentration, we co‐transfected cells with plasmids expressing *Nm*PAL from different promoters. Besides the strong cytomegalovirus (CMV) promoter, we used the medium‐strength ubiquitin C (Ubc) and the weak metallothionein I (MT) promoter with relative expression strengths of 39% and 3%, as quantified by flow cytometry and normalization to CMV‐driven mCherry‐*Nm*PAL levels. Next, we assessed the light‐dependent control of EGFP expression as before by flow cytometry (**Figures** **4a**,3c). *Nm*PAL expression driven by the Ubc promoter led to light‐dependent downregulation of EGFP expression under the control of optozyme OzS4, but only a weak regulation occurred using the OzS5 construct (Figure 4b). Of note, no EGFP repression was observed in darkness for Ubc‐driven *Nm*PAL expression, and OzS4‐controlled EGFP expression in darkness was at similar levels as those observed for HHR(m) (Figure 4b). When using the MT promoter, only minor light dependence of EGFP expression was observed (Figure 4b). Using a HEK cell line stably expressing mCherry‐*Nm*PAL (HEK293PAL),^[^ ^11^ ^]^ we observed similar results to those obtained from transient transfection of the Ubc‐promoter driven constructs (Figure 4b,c and Supporting Figure S2f, Supporting Information). mRNA expression levels, measured by Q‐PCR, corresponded with light‐regulated EGFP levels (Figure S2g, Supporting Information). Using a photomask, we show a spatial reduction of EGFP expression with the optozyme OzS4, but not with the non‐binding variant OzS4M21 (Figure 4d; Figures S4 and S5, Supporting Information).

### 2.4 Adaptability of Motif 3 to shRNA‐ and CRISPR/dCas9‐Based Approaches to Regulate Gene Expression

**Figure 5:**
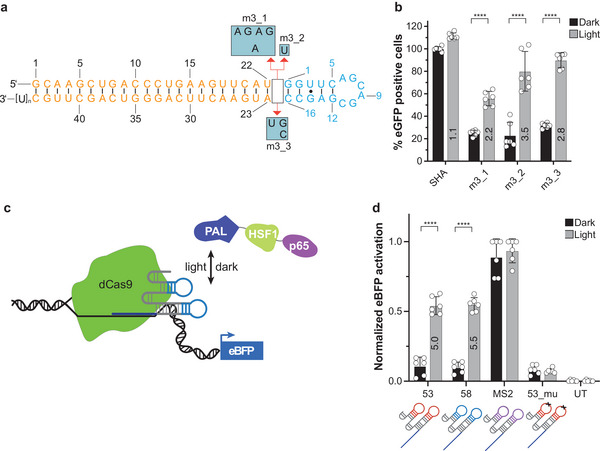
Validation of motif 3 as an optoribogenetic tool in mammalian cells. a) Motif 3 (blue) as an apical loop domain in shRNAs was tested in conjunction with structural variations (m3_1 to m3_3) in the hinge region (blue boxes) and a siRNA (orange) targeting EGFP. b) Fraction of cells expressing EGFP after transfection with the indicated shRNA. Values are normalized to SHA, which was incubated in darkness. Grey bars: light conditions, black bars: dark conditions. Values are means ± s.d. Fold changes calculated from light versus dark conditions are represented by the numbers in the grey bars. Grey bars: light conditions, black bars: dark conditions. Values are means ± s.d. c) Light‐driven transcription activation based on the dCas9 and PAL‐aptamer system.^9^ Gene expression is activated under light by conditional recruitment of transcriptional activators (HSF1, p65) to the target gene promoter through the light‐dependent interaction of the PAL fusion protein with the motif 3 aptamer (58 in d, blue sites) embedded in the respective guide RNA. d) Light‐dependent induction of EBFP expression using sgRNAs with embedded the PAL‐binding hairpin motif 2 (53) or motif 3 (58) or a non‐binding mutant (control). Grey bars: light conditions, black bars: dark condition. The data is presented as mean values ± s.d. after normalization to the value obtained from the MS2‐aptamer system in light/dark using min‐max scaling. Fold changes calculated from light versus dark conditions are represented by the numbers in the grey bars. The respective schematic of the sgRNAs is shown below the bars. Colors indicate the site in which the aptamers were incorporated, *x* indicates the mutation. b) *n* = 3, in duplicates. c) *n* = 2, in triplicates. ^****^: *p* ≤ 0.0001; all other light/dark differences were found to be non‐significant.

Having demonstrated that motif 3‐modified optozymes enable the light‐control of gene expression in mammalian cells, we next evaluated whether this motif can be applied more broadly, e.g., for regulating transcription initiation and RNA interference as previously shown for motif 2.^[^ ^9^, ^11^ ^]^ We constructed short hairpin (sh)RNA chimeras built from an EGFP‐targeting siRNA and motif 3 (**Figure** **5**a). We designed chimeric variants with three different hinge regions as these have been found to impact shRNA light‐dependent regulation.^[^ ^11^ ^]^ Expression of these chimeras in mammalian cells demonstrates the capability of motif 3 to regulate shRNA/siRNA activity by light (Figure 5b,c). A control RNA that binds *Nm*PAL but does not target EGFP showed no reduction of gene expression nor light‐dependency. Recently we demonstrated activation of gene expression with dCas9 and appropriately designed guide (g)RNAs that recruit a fusion protein of *Nm*PAL, HSF1, and p65 in a light‐dependent manner.^9^ By embedding motif 3 in the hairpin and stem loop regions of dCas9 gRNAs (Figure 5c), we also achieved recruitment of the PAL fusion protein to target DNA loci (Figure 5c), thereby inducing gene expression in a light‐dependent manner to a similar extent as we have previously shown for motif 2^[^ ^9^ ^]^ (Figure 5d; Figure S6, Supporting Information). Taken together, these data illustrate that motif 3 is a broadly applicable synthetic biology tool enabling the optoribogenetic regulation of gene expression by light on different levels of gene expression.

## 3 Conclusion

In summary, we describe a high‐affinity PAL‐binding RNA hairpin, termed motif 3, that was identified by a combination of SELEX and genome mining. Motif 3 is widely applicable in synthetic biology and can be used as a novel optoribogenetic tool in bacteria and mammalian cells for different modalities. Compared to motifs 1 and 2, motif 3 shows tighter RNA interactions and more pronounced regulation by light. The superior performance and broad suitability of the motif 3‐aptamer are further supported by a recent report on the regulation of gene expression in bacteria.^[^ ^27^ ^]^ Importantly, choosing the correct motifs for light‐regulation by *Nm*PAL seems to be critical: motifs 1 and 3 showed the best performance in bacteria but in mammalian cells, it was motifs 2 and 3. As reported by us^[^ ^26^ ^]^ and more recently by others,^[^ ^35^ ^]^ motif 1 leads to comparatively poor performance in mammalian cells. Although the three motifs (1‐3) display sequence and topological similarities, the molecular basis and environmental factors responsible for the distinct activities remain elusive and require further analysis. Nevertheless, introducing motif 3 species independently allows for comparable optoribogenetic studies. Regardless of the host system, the precise optoribogenetic scenario, and the motif employed, it is imperative that *Nm*PAL be unmodified at its C‐terminus. This principle is well documented in the original report on *Nm*PAL, and any departure from it, as recently conducted,^[^ ^35^ ^]^ is bound to negatively affect the degree of light regulation attainable.

The motif 3 aptamer facilitated the introduction of the concept of optozymes, i.e., blue light‐control of hammerhead ribozyme activity by *Nm*PAL, which can be harnessed for genetically encoded spatiotemporal regulation of gene expression. While OzS5 was found to be more effective in bacteria, OzS4 was the most efficient optozyme in mammalian cells. In bacteria, the *Nm*PAL‐bound state prevents HHR self‐cleavage, thereby keeping the Shine‐Dalgarno sequence inaccessible and gene expression blocked. In mammalian cells, the *Nm*PAL‐bound state by contrast induced optozyme self‐cleavage and thereby repressed expression. Intriguingly, we observed different activities of OzS4 and OzS5 in mammalian cells, an unexpected trait that however correlates with findings on other aptazymes.^[^ ^36^ ^]^ In our case, the disparate traits seem to be mainly attributable to different self‐cleaving properties of the two optozymes in mammalian cells. While OzS4 self‐cleavage function is activated by interacting with *Nm*PAL, OzS5 seems to be constitutively active in the absence of *Nm*PAL. At elevated concentrations of *Nm*PAL, OzS4 shows cleavage activity also in darkness, but this activity was significantly diminished by tuning the *Nm*PAL concentration. In the case of the OzS5 optozyme and high *Nm*PAL concentrations, the reporter gene experiments reveal an induction of gene expression upon irradiation. This effect might be explained by intermingled contributions of self‐cleavage and the protection of the 3′‐end of the cleaved mRNA by *Nm*PAL, which may remain bound to the mRNA after optozyme cleavage and thus may cause delayed degradation. This effect is not observed in OzS4, which we attributed to the less intense interaction between OzS4 and *Nm*PAL compared to OzS5 (see Figure S2d, Supporting Information).

Taken together, the optozyme OzS4 demonstrates effective regulation, allowing high levels of gene expression in darkness (comparable to those observed from 3′‐UTR embedded cleavage‐deficient HHR mutants) and successful suppression of gene expression upon irradiation to background levels (comparable to those observed for 3′‐UTR embedded HHR). A specific advantage of this approach and the *Nm*PAL: aptamer system, in general, is that it acts at the RNA level,^[^ ^37^ ^]^ thus rendering it orthogonal to the majority of strategies for signal‐dependent gene regulation.

## 4 Experimental Section

### Genome Mining

To identify the putative native RNA sequence interacting with the PAL photoreceptor protein, the motifs obtained by NGS and mutation analysis from the previous publication were harnessed.^[^ ^26^ ^]^ In a first attempt, motif 2 was searched in the genome of *Nakamurella multipartita*, yielding no results. In a second attempt, a Java program was written, which used regular expression to screen for putative native hairpins. Thereby, the consensus sequences including the mutation analysis (S = G/C, W = A/U, V = A/C/G) and the predicted secondary structure of motif 2 were used as a starting point. A search pattern was generated, which included formation of variable stem base pairs X‐Y including an U‐G wobble base pair and a loop comprising nine nucleotides. Out of these loop nucleotides, six nucleotides remained unmodified, whereas positions 4, 7, and 9 (N_1_, N_2_, N_3_) could either be an A, U, G, C, or deletion. Hit sequences were analyzed via Mfold^[^ ^38^ ^]^ for the most likely correct folding of the hairpin in the context of the RNA strand, in which the hairpin is embedded. Correctly folding hit sequences were further analyzed whether they were located in the untranslated region of the genome, as assumed for ANTAR RNA sequences via BLAST. Out of the hit sequences, only Nm60 fit these requirements. Nm60 bears a deletion at loop position 9, resulting in additional base pairing of loop nucleotides and subsequently formation of a hairpin with only six loop nucleotides according to Mfold prediction. During the last step, the NGS data of the PAL selection was analyzed for the appearance of Nm60 as UUCAGCAGCGAG. The analysis yielded enriched sequences of the newly found motif 3, which were forming hairpins according to Mfold. The sequence with the highest frequency was termed aptamer 58. No matches were found for motif 1 using the method described above.

### Cloning for Bacterial Activation Experiments

The plasmid for the bacterial reporter assay was constructed based on the plasmids pCDF‐PAL and pET‐28cDsRed‐SP.^[^ ^26^ ^]^ Briefly, the *Nm*PAL and *Ds*Red genes were combined in one pCDF plasmid and put under constitutive promoters. Then, the control hammerhead ribozyme (HHR) sequence^[^ ^17^ ^]^ was introduced by PCR and blunt‐end ligation into the 5′ untranslated region of the *Ds*Red gene, with the Shine‐Dalgarno (SD) embedded in it, generating the plasmid pCDF_NmPAL_HHR. This plasmid was then used as a template to construct the different aptamer variations by PCR amplification and blunt‐end ligation. The used sequences were provided in Tables S1 and S2 (Supporting Information).

### Protein Expression and Purification of *Nm*PAL

Protein expression and purification of *Nm*PAL‐his, bearing a C‐terminal hexahistidine tag, and *Nm*PAL were carried out as previously described.^[^ ^26^, ^27^ ^]^

### Fluorescence Anisotropy

The binding of *Nm*PAL to RNA was measured by fluorescence anisotropy, using 5′ TAMRA‐labeled 58 (5′‐ACCUUCGGUUCAGCAGCGAGCCG‐3′) and 58.M21 (5′‐ACCUUCGGUUCAGCUGCGAGCCG‐3′).^[^ ^27^ ^]^ Briefly, 4 nm labeled RNA were incubated with different concentrations of *Nm*PAL in darkness in 12 mM HEPES/HCl pH 7.7, 135 mm KCl, 10 mm NaCl, 1 mm MgCl_2_, 10% w/v glycerol and 100 µg/mL BSA. Fluorescence anisotropy was first measured in darkness. Then, the plate was illuminated with blue light (465 nm, 5 mW cm^2^) for 60 s, and the fluorescence was recorded again. All measurements were performed using a multi‐mode microplate reader (CLARIOstar) at (540 ± 10) nm excitation and (590 ± 10) nm emission. The light intensity was measured using a power meter (model 842‐PE, Newport) and a silicon photodetector (model 918D‐UV‐OD3, Newport), and the data were analyzed using Fit‐o‐mat. For 58M21 and 58 aptamers in dark‐adapted state, the data were fitted to single‐site binding isotherms Equation 1).

(1) $$r\;=\;r_{0}+r_{1}\frac{\left[\mathrm{NmPAL}\right]}{\left[\mathrm{NmPAL}\right]+\;K_{D}}$$

In the case of 58 under light conditions, the data were fitted to a model with two independent sites. Given that certain dissociation constants were in the same range as the PAL concentrations used, a modified single‐site model was employed to account for the free *Nm*PAL depletion Equation (2)

(2) $$\begin{array}{ccc} r\; & & =r_{0}\;+\;\frac{r_{1}}{2}\times \left\{1+\frac{\left[\mathrm{NmPAL}\right]}{c_{total}}+\;\frac{K_{D}^{1}}{c_{total}}\right.\\ & & \left.-\;\sqrt{{\left(1+\frac{\left[\mathrm{NmPAL}\right]}{c_{total}}+\;\frac{K_{D}^{1}}{c_{total}}\right)}^{2}-4\frac{\left[\mathrm{NmPAL}\right]}{c_{total}}}\;\right\}+\frac{r_{2}}{2}\\ & & \times \left\{1+\frac{\left[\mathrm{NmPAL}\right]}{c_{total}}+\;\frac{K_{D}^{2}}{c_{total}}\right.\\ & & \left.-\;\sqrt{{\left(1+\frac{\left[\mathrm{NmPAL}\right]}{c_{total}}+\;\frac{K_{D}^{2}}{c_{total}}\right)}^{2}-4\frac{\left[\mathrm{NmPAL}\right]}{c_{total}}}\;\right\} \end{array}$$

where *c*_total_ is the total RNA concentration in the assay.

### Bacterial Reporter Gene Assay

The light‐dependent gene expression controlled by the binding of *Nm*PAL to HHR was evaluated in a reporter‐gene system. *E. coli* CmpX13 cells were transformed with the pCDF_NmPAL_HHR reporter plasmids. Bacterial starter cultures were grown in LB medium (500 µL) containing streptomycin (100 µg mL^−1^) in 96‐well deep blocks at 37 °C for 16 h. Then, two identical 96‐well clear microtiter plates containing LB medium (198 µL), starter culture (2 µL), and streptomycin (100 µg/mL) were prepared, sealed, and incubated at 37 °C for 24 h in darkness or under blue light (465 nm, 25 µW cm^−2^), respectively. The light intensity was determined the same way as in the fluorescence anisotropy assay, but also taking into account the attenuation caused by the breathable seal. The optical density at 600 nm and fluorescence of *Ds*Red (excitation wavelength of (554 ± 9) nm and emission wavelength of (591 ± 20) nm) were measured using a Tecan M200 plate reader. Data were normalized and represented the mean ± standard deviation of three biologically independent replicates.

### Molecular Biology for Experiments with Mammalian Cells

All oligonucleotides were purchased from Ella Biotech, Planegg, Germany. The hammerhead ribozyme (HHR) sequence, the cleavage deficient mutant, and the optozymes were incorporated into pEGFP‐N1 (Clontech) in the 3′ UTR of the EGFP coding sequence (CDS) via the *NotI* restriction enzyme site. Plasmid pIRESneo‐FLAG/HA Ago2 was kindly provided by Thomas Tuschl. The plasmids pmCherry‐C1 and pEGFP‐N1 were purchased from Takara Clontech. The sequences used for the mammalian HHR experiments are listed in Table S3 (Supporting Information). All miR and shRNAs used in this study were cloned in the pSilencer 2.0‐U6 plasmid backbone using restriction cloning. Corresponding miR and shRNA sequences were listed in supplementary (Table S5, Supporting Information). The sgRNA containing aptamer 58 was designed using the SG9 sgRNA scaffold as a template. The sgRNA 58 insert was constructed by hybridizing a sense and antisense oligonucleotide (Table S6, Supporting Information). The SG9 scaffold in the pENTR.hU6.SG9 plasmid was replaced by the hybridized sgRNA 58 insert using AQUA cloning as previously described.^[^ ^5^ ^]^ Sequence and schematic of sgRNA 58 is provided in Figure S6 (Supporting Information).

### Mammalian Cell Culture

HEK293T cells were cultured in DMEM, high glucose, GlutaMAX supplemented with 10% FCS at 37 °C, and 5% CO_2_. The HEK293PAL cell line was cultured in DMEM, high glucose, GlutaMAX supplemented with 10% FCS, 1% non‐essential amino acids (NEAA), 1% sodium pyruvate (Thermo Fisher Scientific) and 400 µg mL^−1^ G418 (Gibco) at 37 °C, 5% CO_2_. HeLa cells (CLS, Eppenheim, Germany) were cultured in DMEM (Gibco Thermo Fisher Scientific, Waltham, USA) supplemented with 10% fetal calf serum (FCS, Sigma‐Aldrich), 1% non‐essential amino acids (NEAA) and 1% Sodium Pyruvate (Thermo Fisher Scientific). The cell lines were sub‐cultured every 2–3 days according to standard cell culture protocols. The cell lines were regularly tested for Mycoplasma contamination using VenorGeM Classic (Minerva Biolabs).

### LED Array for Mammalian Cell Culture Experiments

Blue light was administered to cells in pulses (30 s light on, 30 s light off) by a custom LED array with *λ*_max_ = 465 nm and adjustable light intensity. The LED array was powered using a custom‐built microcontroller. Cells were exposed to light immediately after transfection until they were subjected to further investigation.

### Flow Cytometry Experiments with Mammalian Cells

For the analysis of light‐dependent EGFP expression in the HHR constructs, 6 × 10^4^ HEK293T cells were seeded on poly‐L‐lysine coated 48‐well cell culture plates. The next day, cells were transfected according to the manufacturer's instructions using pEGFP reporter gene plasmid (100 ng) and mCherry/mCherry*Nm*PAL (400 ng) bearing effector plasmid with Lipofectamine 2000 (Thermo Fisher Scientific) in OptiMEM for 4 h. Subsequently, OptiMEM was replaced by DMEM, high glucose, GlutaMAX supplemented with 10% FCS and cells were incubated in presence of blue light (34 µW cm^−^^2^) or darkness at 37 °C, 5% CO_2_ for 24 h. Before the measurement, the cells were detached using TrypLE Express Enzyme (1X) (80 µL), without phenol red (Gibco) for 10 min. Cells were re‐suspended in DPBS (100 µL) and transferred into a 5 ml round bottom polystyrene test tube (Falcon) and centrifuged at 300 x *g* for 1 min. Afterwards the cells were analyzed by flow cytometry. The relative expression of mCherry*Nm*PAL using different promoters was evaluated by exploiting the intrinsic fluorescence of PAL in darkness. HEK293T cells were transfected with pmCherryPAL, pUbc‐pmCherryPAL, or pMT‐mCherryPAL (400 ng) as described for the EGFP expression in the HRR constructs. The resultant geometric mean fluorescence intensity (gMFI) values were normalized to the fluorescence of untransfected cells and mCherryn*Nm*PAL transfected cells. For the shRNA experiments, 1 × 10^5^ HEK293PAL cells were seeded in two 24‐well plates per well and incubated in darkness for 24 h. During the transfection, 1.5 µL Lipofectamine2000 (Thermo Fisher Scientific) and 250 ng plasmid DNA (e.g., pSilencer, AGO2 and pEGFP‐N1 plasmid at a mass ratio of 2:2:1) was used per well. Plasmids and Lipofectamine 2000 were each diluted in 50 µL Opti‐MEM (Thermo Fisher Scientific) per well and incubated 5 min before mixing at 20 °C. After 20 min of incubation at 20 °C, 100 µL transfection mix was added to each well. 4 h after transfection, 60 µL FCS were added per well. Cells were incubated for 44 h in the presence of blue light (106 µW cm^−^^2^) using the LED array or in darkness. Then, the cell supernatant was aspirated, cells were washed and resuspended in DPBS (25 °C). The subsequent analysis was performed using flow cytometry. For CRISPR/dCas9 based activation experiments, HeLa cells were seeded at 7 × 10^4^ cells per well in 24‐well plates and cultured for 24 h in the cell culture incubator. For each transfection, four plasmids were mixed in a ratio of 2:1:1:1 (dCas9:Effector:sgRNA:EBFP reporter) yielding a final amount of 200 fmol. The plasmids and the detailed experimental procedure were described previously.^[^ ^5^ ^]^

### Flow Cytometry Analysis

Flow cytometry was performed on a BD FACS Canto II (BD Biosciences). Data was processed using FlowJo version 9.6.3 software. At least 30.000 cells were analyzed from each sample. The individual gating strategies are depicted in Figure S3 (Supporting Information).

### Photomask Experiment

HEK293 cells (2.5 × 10^5^) stably expressing mCherry‐PAL were seeded in gelatine coated black 24‐well plates with clear bottom (VisionPlate, 4titude). After 24 h, the cells were transfected according to the manufacturer's instructions using pEGFP reporter gene plasmid (100 ng) with Lipofectamine 2000 (Thermo Fisher Scientific) in OptiMEM for 4 h. The medium was replaced by the cultivation medium. A plus‐shaped photomask was applied to the bottom, which masked of half of the wells in the plate, whereas the other half of the plate was completely shielded from light. The cells were incubated in presence of constant blue light (34 µW cm^−^^2^) or darkness at 37 °C, 5% CO_2_ for 24 h. Afterward, the wells were analyzed using a confocal laser scanning microscope (LSM 710), using a 10 × /0.45 objective and image concatenation (10% overlay). Each well was imaged as tile scan with 12 × 12 tiles. Images were processed using Zen 3.4 (Blue Edition, Zeiss). eGFP fluorescence was visualized as green color and mCherry as red color. Image brightness was adjusted to + 50 and images were adjusted using Adobe Photoshop CS5 software.

### mRNA Isolation from Mammalian Cells and qPCR

mRNA isolation was performed using Magnetic mRNA Isolation Kit (New England Biolabs). Briefly, 5 × 10^4^ cells were lysed using Lysis/Binding Buffer and the suspension was mixed with 50 µL Oligo d(T)_25_ beads for 10 min under agitation at room temperature. The beads were pulled using the magnetic rack and the beads were washed according to the manufacturer's instructions. The mRNA was eluted with elution buffer (50 µL) at 50 °C for 2 min. Subsequently, the isolated mRNA was reverse transcribed using Maxima H Minus reverse transcriptase (Thermo Scientific) containing 50 ng mRNA, 5 µm Oligo d(T)_18_ primer (5′‐TTTTTTTTTTTTTTTTTT‐3′), 20 U RNasin recombinant ribonuclease inhibitor (Promega), and 200 U Maxima H Minus RT in a total volume of 20 µL 1x First‐Strand Buffer at 50 °C for 30 min. Reverse transcription was terminated by heating samples at 85°C for 5 min. Prior to qPCR, cDNA was treated with 5 U RNase H (New England Biolabs) at 37 °C for 30 min and the enzyme was inactivated at 65 °C for 20 min. For qPCR, samples were diluted 1:500 and reactions were set up using Luna Universal qPCR Master Mix (New England Biolabs) according to the manufacturer's instructions. Thereby, the EGFP gene was monitored using the forward primer sequence 5′‐AAGGGCATCGACTTCAAGG‐3′ and the reverse primer sequence 5′‐TGCTTGTCGGCCATGATATAG‐3′. As an internal control, the GAPDH gene was selected using the forward primer sequence 5′‐GTCTCCTCTGACTTCAACAGCG‐3′ and the reverse primer sequence 5′‐ACCACCCTGTTGCTGTAGCCAA‐3′. The qPCR was performed on a CFX96 Touch Real‐Time PCR Detection System (Biorad) with initial denaturation at 95 °C for 60 s following 30 cycles of denaturation at 95 °C for 15 s and extension at 60 °C for 30 s + readout. The Δ*C*_q_ value was determined as following Δ*C*_q_ = *C*_q_ (*EGFP*) − *C*_q_(*GAPDH*). To determine the difference between light and dark the ΔΔ*C*_q_ was calculated as follows Δ*C*_q_ =  Δ*C*_q_(*light*) − Δ*C*_q_(*dark*).

### RiboGreen Interaction Assays

The *Nm*PAL protein was biotinylated using a fourfold molar excess of EZ‐Link‐*sulfo*‐NHS‐LC‐Biotin (Thermo Fisher Scientific) according to the manufacturer's instructions. Non‐reacted biotin was removed using Zeba Spin Desalting Columns, 7K MWCO (Thermo Fisher Scientific). Immobilization was performed with biotinylated *Nm*PAL (100 µL) in ICB (12 mm HEPES pH 7.4, 135 mm KCl, 10 mm NaCl, and 10% Glycerol) with a final concentration of 1.25 µm at 4 °C overnight in the dark. Indicated RNAs were incubated in ICB (100 µL) in a final concentration of 500 nm for 30 min at 29 °C in light (465 nm, 2.15 mW cm^−2^) or darkness. Subsequently, the samples were washed 3x with ICB (200 µL). For the detection of bound RNA, 150 µL detection buffer (Quant‐iT RiboGreen RNA Reagent diluted 1:500 in 1x TE (10 mm Tris‐HCl pH 7.5, 1 mm EDTA)) was added and incubated for 1 h at room temperature in the dark. The fluorescence was measured using the Tecan Ultra plate reader with λ_ex_ = 500 nm and λ_em_ = 525 nm. The sequences used for the RiboGreen interaction assays are listed in Table S4 (Supporting Information).

### Statistical Analysis

Data were analyzed, employing either one‐way or two‐way analysis of variance (ANOVA). In case of statistically significant ANOVA results, either a Dunnett's‐ (one‐way ANOVA) or Tukey (two‐way ANOVA) post hoc tests were conducted. Irradiation and genotype (i.e., plasmids transfected) were applied as main factors for the two‐way ANOVA. If the data were not normally distributed, we applied non‐parametric tests for the analysis (Brown‐Forsythe ANOVA test). Analysis was done by using the GraphPad Prism version 9 for MAC. Unless otherwise indicated, data are expressed as mean ± SD, and statistical significance was considered when *p* ≤ 0.05. Significance levels are indicated as follows: ^*^ *p* ≤ 0.05, ^**^ *p* ≤ 0.01, ^***^ *p* ≤ 0.001, ^****^ *p* ≤ 0.0001.

## Conflict of Interest

The authors declare no conflict of interest.

## Supporting information

Supporting Information

## Data Availability

The data that support the findings of this study are available from the corresponding author upon reasonable request.
