# Supplementary material for: An RNA Motif That Enables Optozyme Control and Light‐Dependent Gene Expression in Bacteria and Mammalian Cells
Source: Adv Sci (Weinh). 2024 Jan 16;11(12):2304519. doi: 10.1002/advs.202304519 (PMC10966536; doi:10.1002/advs.202304519)
Supplement: Supplementary file 1 — Supporting Information [file ADVS-11-2304519-s001.pdf]

ADVANCED SCIENCE
Open Access

Supporting Information

for *Adv. Sci.*, DOI 10.1002/advs.202304519

An RNA Motif That Enables Optozyme Control and Light-Dependent Gene Expression in Bacteria and Mammalian Cells

*Georg Pietruschka, Américo T. Ranzani, Anna Weber, Tejal Patwari, Sebastian Pilsl, Christian Renzl, David M. Otte, Daniel Pyka, Andreas Möglich and Günter Mayer**

Supporting Information

An RNA motif that enables optozyme control and light-dependent gene expression in bacteria and mammalian cells

*Georg Pietruschka[1,a], Américo T. Ranzani[2], Anna Weber[1,3], Tejal Patwari[1], Sebastian Pilsl[1], Christian Renzl[1,3], David M. Otte[1], Daniel Pyka[1], Andreas Möglich[2], Günter Mayer[1,3,]**

```
5′GGCGACCCCCTGGTTCTGCTGGCCGGCCCGCGGCAGGCCGGGCACGCAGAGTGGCCCGGTTCCCCGAATGC
GCCGGTCATCGGATCCGCTTGCCGTGCATGTCGCCGTGATGATCGGGAACAGGGTCATTGGTGGGCGGTGCGC
GCGCGATCACCGTCCATCCGGATGCCCGGCCGGTTCAGCAGCGAGCCGGCCGGGTCATACCAATCACCAGATG
CGAGGCTGGAGTCGTGAACGGGCCGGACGCAACTGCCGACGAGGATGGAATCGACGCCGGGGTGGTCGGTGAC
GCGTCCGTGCTGGCCCGGGTGTTCGAGGACTCGCCGGCCATTCACGTCGCCCTGCAGGGACCCGAGCACACCA
TCGTCGCCATCAATGCGGTGACCCGGCGGCTGCTGGGGGATCGCCCGACGCTCGCCCGGCCGTGTGCGGCCGC
CATCCCCGAACTCATCGGCCGGTCATTGCTGGACCGCCTGGACGAGGTCTACGCCTCCGGTCAGCCCATCACC
GCCCGGGATCGGCGGGTGCACCTGGACGGCGGCGCCGAGGACGAGATCGACCTGGAGTGGTTCGTCACCTTCA
TCATCTCGCCGTGGCGGTACGCCGACGGCACGATGCGTGGCGTCATCGTGCAGGCGGTGGACACGACCTCGCA
GCTGCAGGCCCGCCGCCAGGCCACCCAGAGCGGCAGCGAGTTCCGCCGCCGGTACCGGGCCGCCCTGGACGTC
ATCGACGAGCTGCAACGCACGTTGCTGCCCTCCAGCCTGCCGGTGCTGCCGCAGCTGGAGCTGGCTGCCCGGT
ACCTGCTGGCCGACGTCGAGCACGCCGCCGGCGGCGACTGGTTCGACGGGTTCATGCTGGCCGACCACCGGCT
GGCCGTGGTGGTCGGCGACGTCGTCGGGCACGGGGTGGCCGCGGCCGGGGTGATGAGCCAGCTGCGGTCGGTG
CTGTCGTACCTGTTGGACACCGGGGTGGAGCTGGCCGAGGCGGTCACCCAGCTCGACCGGTTCGCCTCCCGCA
CGCCGGGTGGGTTCGCGGCCACCGTGTGCGTCGTGCTGGTCGAACCGGCCAGCGGCGCGATGACGTACGTGAC
CTGCGGGCATCCCGCGCCGATGGTCGTCGACGCGGACGGGGTGGCCCGATTCCTGCCGCTGTCCGGGCACCGG
CCGCTGGGCGCCGGAGCGTCCGCCGCGCCGATCACCGATTCGCTGGGGATCGGGGAGGTGCTGGTGCTGTTCA
GCGACGGTCTGGTCGAGCGGCCGGATCGGACCTGGGAATCGGGCCTGGCGCTGCTGGCCACCATCACCGGGGA
CGCGGTCCTGGGCCGCGACCTGCCGGAGGGTTCACCCGAGTCGGTCCCCGAACGGGTGTGCGCCCACGCCGTG
GAGATGATGACCCGCCCGGGCCACCTGGACGACGTCACCATGGTCGCGGTGCAGCGGCGCGCGGTGCCGGTCG
AGCCCTGGTACGCCGAGTACCCGGCCCGAGCGGACCGGTTGGAGCTGATCCGGGCCGATCTGCGCAGGTGGCT
GCGGGAACGGATGGTCTCCGCCGAGGACGAGACCGCGCTGACCCTGGCGGTCAGCGAGGCGGCGGCCAACTGC
ATCGAGCACGCGTACCGGGACGGTGACGAGGGCACGATCCGGATCCAGGTGAACCTGACCACCAAGGGTGACG
CCTTGCTGGTGGTCGCCGACGAAGGCGGGTGGCGGGACCCGATCACCAAGGCCGGTACTCGAGGCCGGGGCCT
GGCCATGGTGGCCGACGCGGTCGACGACTTCTCGATCGACACCGAGACCGGGGGCACCGTGGTGCGGATGCGG
CGGCGGTTGCACCGGCCGGTCGGCATGCTCGCCGGAGACGGCGTGGTCGAGGTCGGCAGCGCCGAATTCCGGG
CCGAACCGTCCGGGCCGATCGGGGCCCGGGTCATCACCGTTTCCGGCTCGGTCGACCTGGCCTCGGTGCCGGT
GCTGCGCCAGGAGGTGCTGCGGGCCGGCCGCGGCGGCGAGCTCCCGGTGACCATCGACCTGACCGCGGTGACC
CACCTGAGCAGCGCCGGGGTGCAGATGCTGCACACCCTGTCTGCGACCGGGCAGCCGGGCGCGTTGCGGCATC
CGCCGGTGCTGATCGCCGCGTCCGGATCACCGGCGGCCTACGTGCTGGACCTGGCCGGGCTGGTCCGGACCAG
TGACGTCACCGACCGAAGCGATCGAGCCGACCGGCACGACTGA 3′
```

Figure S1. Genome sequence showing motif 3 (green) in the 5’UTR of the PAS/PAC Proteins (blue, Gene Bank: ACV78465.1). Red: putative start codon.

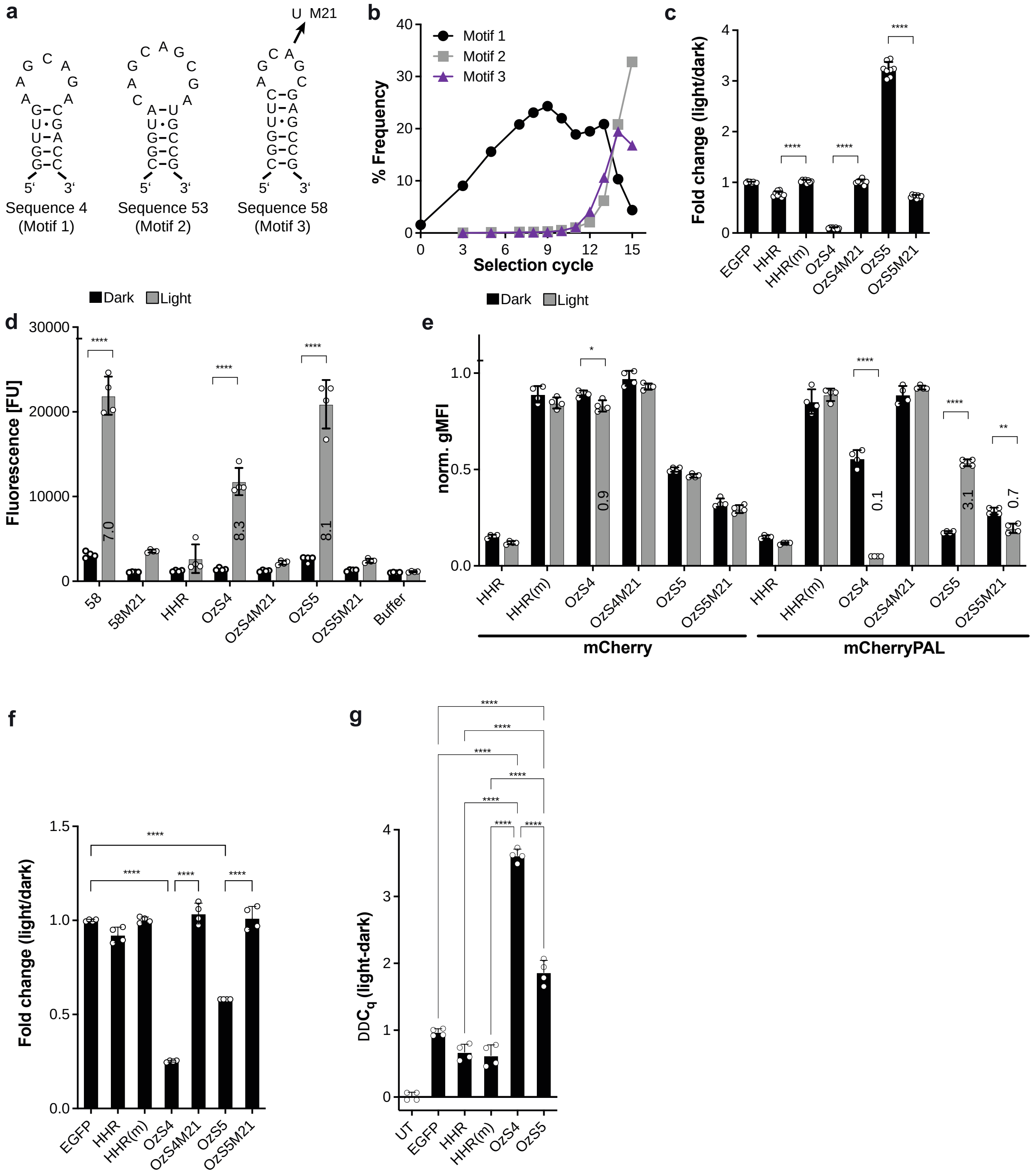

Figure S2. **a)** Sequence and secondary structure of sequence 4, sequence 53, and sequence 58, dubbed motif 1, motif 2, and motif 3, respectively. **b**) Frequency analysis of motif 1, motif 2, and motif 3 of the *Nm*PAL selection.[26] **c**) Fold-change regulation of gene expression of the optozymes in HEK293T cells. **d**) Interaction analysis of the self-cleavage deficient optozymes OzS4 and OzS5, the corresponding non-binding variants (OzS4N21, OzS5M21), the self-cleavage deficient hammerhead ribozyme (HHR(m)), motif 3 (58), and its non-binding variant 58M21with *Nm*PAL in light or darkness. **e**) Regulation of gene expression by light of the optozymes in presence (mCherryPAL) or absence (mCherry) of PAL. norm. gMFI: normalized geometric mean fluorescence intensity **f**) Fold changes calculated from light *vs*. dark conditions from (**e**). **g**) Relative gene expression of EGFP under the control of HHR, HHR(m), OzS4 or OzS5 quantified by qPCR. UT: untransfected. $N = 2$, in duplicates. ****: $p \leq 0.0001$; **: $p \leq 0.01$, *: $p \leq 0.05$; all other differences were found being non-significant.

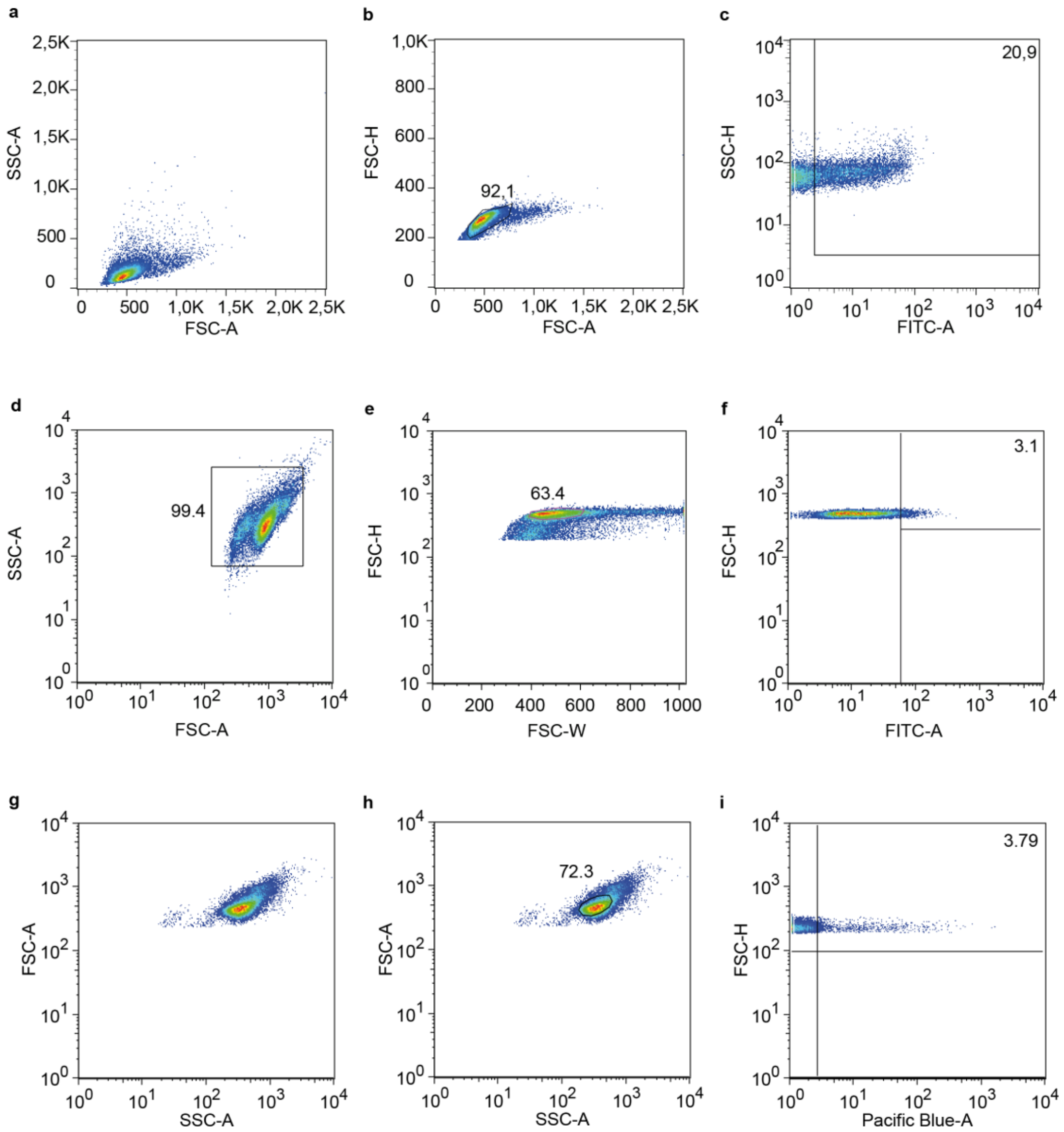

Figure S3. Gating strategies to identify eGFP positive cells for HHR experiments: **a)** 30.000 events were recorded and plotted in an SSC-A vs. FSC-A dot plot. **b)** The main cell population was isolated to exclude cell debris. **c)** The fluorescence threshold of the untransfected population was set to 1% using the quadrant gating method in the FITC-A vs. SSC-H plot. This gating scheme was applied to all the measured samples. Finally, the optozyme activity was elucidated by calculating the geometric mean fluorescence intensity (gMFI) of the threshold fluorescence. Gating strategies to identify EGFP positive cells for shRNA experiments: **d)** Cell debris was excluded using side scatter area (SSC-A) vs. forward scatter area (FSC-A). **e)** Singlet cells were detected using forward scatter height (FSC-H) vs. forward scatter width (FSC-W). **f)** EGFP positive cells were identified using FSC-H vs. fluorescein isothiocyanate area (FITC-A). Cells transfected with SH1 and incubated in darkness were set to 3.1 % EGFP positive cells and gating was applied to all other tested samples. Gating strategies to identify EBFP positive cells for CRISPR/dCas9 experiments. **g)** 30.000 events were recorded and visualized in an SSC-A vs. FSC-A dot plot. **h)** The main cell population was isolated to exclude cell debris. **i)** The background fluorescence of untransfected cells was set to 1% using a quadrant gate. The gating strategy was applied to all samples and the amount of EBFP activation was calculated from the percentage of EBFP positive cells given in the top-right corner. The FSC threshold was set to 5.000. HeLa cells were gated for single cells with an auto fluorescence of 1%. The activation was normalized using the feature scaling method.

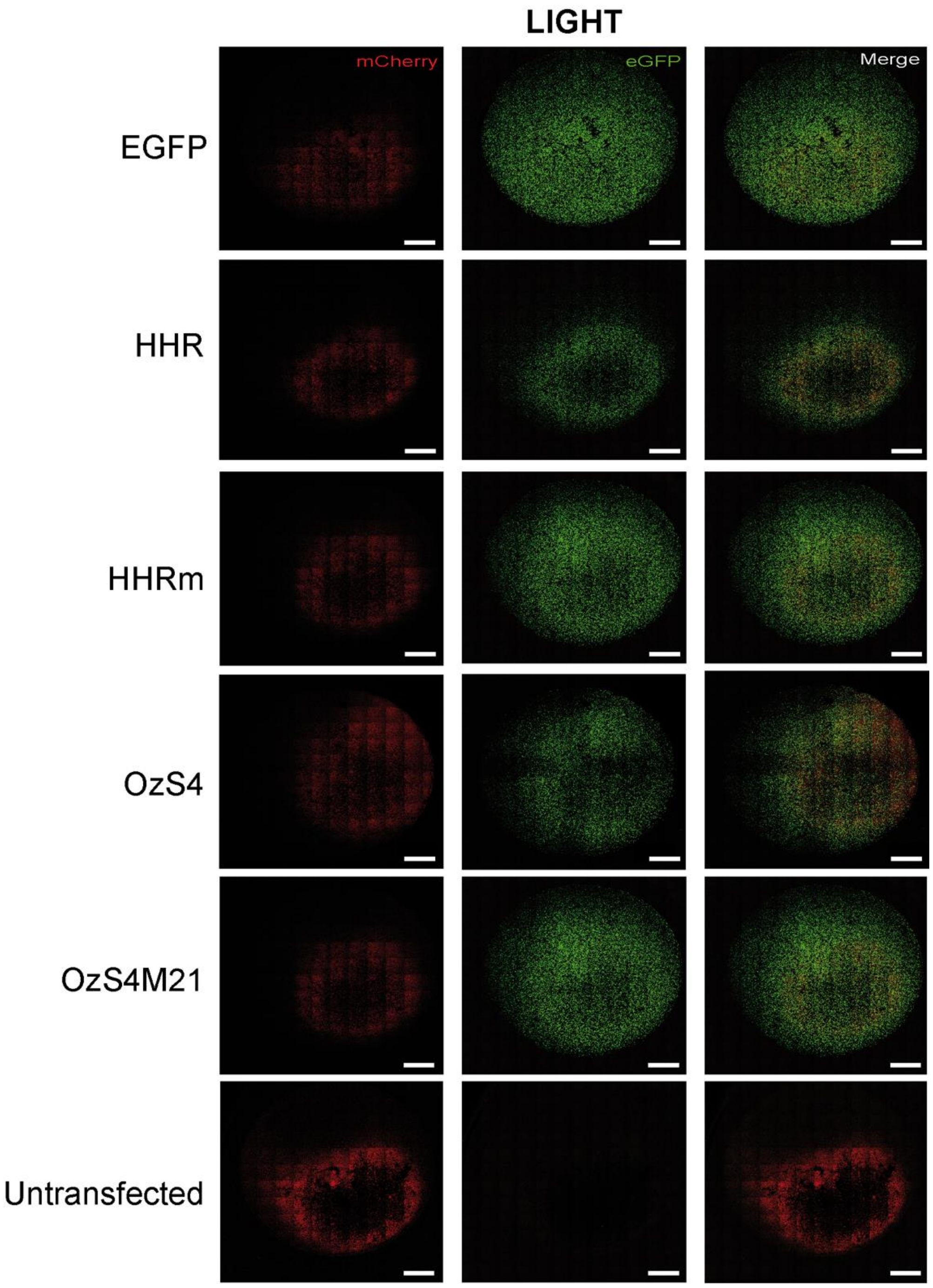

Figure S4. Images from the photomask experiment, where cells were incubated in illuminated conditions. HEK293 cells stably expressing mCherry-PAL were transfected with a EGFP reporter containing HHR, HHRm, OzS4 or OzS4M21. The spatial repression of EGFP, the mCherry signal and a merged variant of the images is shown. White scale bar: 2000 µm. $N = 2$ in duplicates. The brightness of the mCherry and EGFP signal was digitally increased by 50 units to improve the visibility of the EGFP signal. Overall, the mCherry signal was found to be lower compared to the EGFP signal and is more intense at locations on the plate, where the cells tend to accumulate. Dark patches showing low mCherry signal could be due to lower cell densities in those areas.

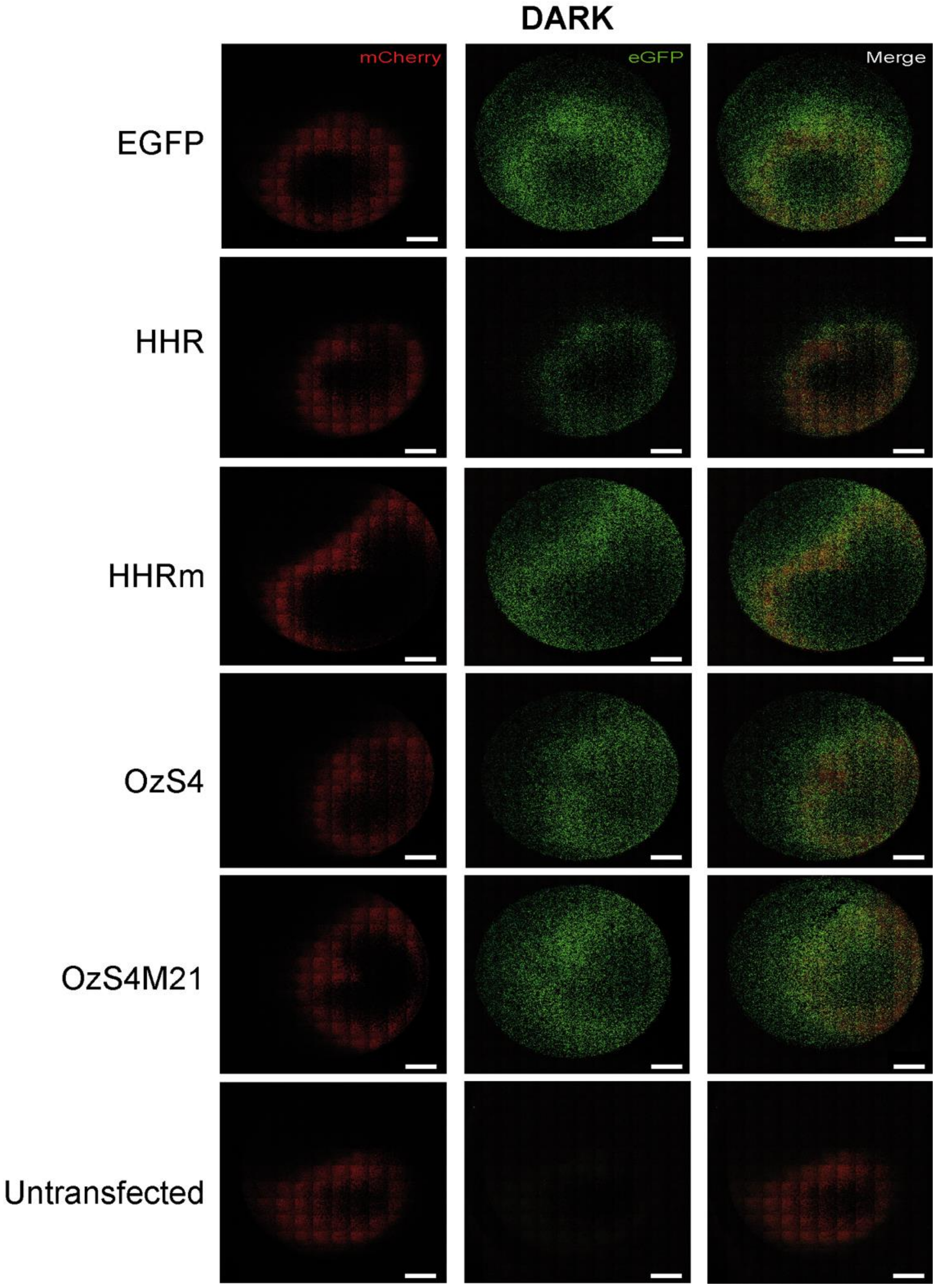

Figure S5. Images from the photomask experiment, where cells were incubated in darkness. HEK293 cells stably expressing mCherry-PAL were transfected with a EGFP reporter containing HHR, HHRm, OzS4 or OzS4M21. The spatial expression of EGFP, the mCherry signal and a merged variant of the images is shown. White scale bar: 2000 µm. $N = 2$ in duplicates. The brightness of the mCherry and EGFP signal was digitally increased by 50 units to improve the visibility of the EGFP signal. Overall, the mCherry signal was found to be lower compared to the EGFP signal and is more intense at locations on the plate, where the cells tend to accumulate. Dark patches showing low mCherry signal could be due to lower cell densities in those areas.

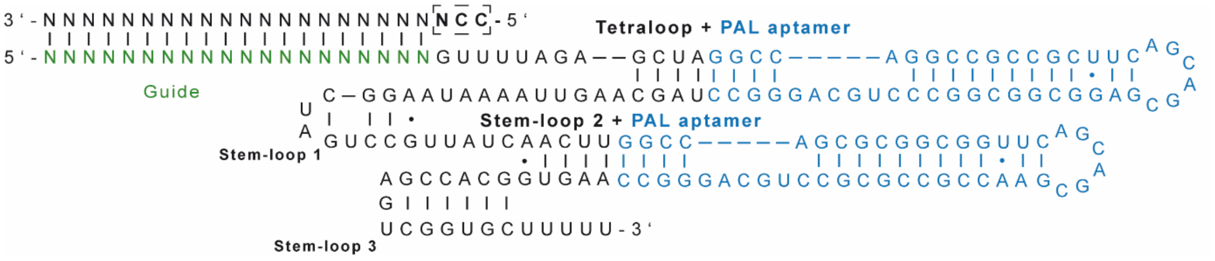

Figure S6. Sequence and design of sgRNA with embedded motif 3.

Tables

Table S1. List of constructs of optozymes in bacterial system. In bold: motif 3 sequence; in bold and red: mutation yielding inactive ribozyme; in bold and gray: M21 mutation; in bold and blue: Shine-Dalgarno sequence.

Construct	Sequence 5´→ 3´
HHR intermediate	UCUCCUUCGGUACAUCCAGCUGAUGAGUCUUCGGUGCGUCCUGGAUUCCACGAAGGAGA
HHR	UCUCCUUCGGUACAUCCAGCUGAUGAGUCCCAAAUAGGACGAAA**CGCGCUUCGGUGCG**UCCUGGAUUCCACG**AAGGAG**A
HHR(m)	UCUCCUUCGGUACAUCCAGCUGAUGAGUCCCAAAUAGGACGA**G**A**CGCGCUUCGGUGCG**UCCUGGAUUCCACG**AAGGAG**A
OzS4	UCUCCUUCGGUACAUCCAGCUGAUGAGUCCCAAAUAGGACGAAA**GUUCAGCAGCGAGC**UCCUGGAUUCCACG**AAGGAG**A
OzS4M21	UCUCCUUCGGUACAUCCAGCUGAUGAGUCCCAAAUAGGACGAAA**GUUCAGCUGCGAGC**UCCUGGAUUCCACG**AAGGAG**A
OzS5	UCUCCUUCGGUACAUCCAGCUGAUGAGUCCCAAAUAGGACGAAA**GGUUCAGCAGCGAGCC**UCCUGGAUUCCACG**AAGGAG**A
OzS5M21	UCUCCUUCGGUACAUCCAGCUGAUGAGUCCCAAAUAGGACGAAA**GGUUCAGCUGCGAGCC**UCCUGGAUUCCACG**AAGGAG**A

Table S2. Oligonucleotides used for cloning of optozymes into the bacterial vector system

Name	Sequence 5´→ 3´
HHR_i_fw	CTTCGGTGCGTCCTGGATTCCACGAAGGAGATATACCATGGGCAGCAGCCATC
HHR_i_rv	ACTCATCAGCTGGATGTACCGAAGGAGATTTAAAGTTAAACAAAATTATTTCTAGAGGGG
HHR_fw	GGTACATCCAGCTGATGAGTCCCAAATAGGACGAAACGCGCTTCGGTGCGTCCTGGATTC
HHR(m)fw	CCCAAATAGGACGAGACGCGCTTCGG
Oz_rv	TTTCGTCCTATTTGGGACTCATCAGC
OzS4_fw	GTTCAGCAGCGAGCTCCTGGATTCCACGAAGGAGATATACC
OzS5_fw	GGTTCAGCAGCGAGCCTCCTGGATTCCACGAAGGAGATATACC
OzS4M21_fw	GTTCAGCTGCGAGCTCCTGGATTCCACGAAG
OzS5M21_fw	GGTTCAGCTGCGAGCCTCCTGGATTCCACG

Table S3. List of optozyme constructs used for expression in mammalian cells. In bold: motif 3 sequence; in bold and gray: mutation yielding inactive aptamer (M21); in bold and red: mutation yielding inactive HHR.

Construct	Sequence 5´→ 3´
HHR	GCGGCCGCGUACAUCCAGCUGAUGAGUCCCAAAUAGGACGAAACGGCGAAAGCCGUCCUGGAUUCCAGGCGGCCGC
HHR (G12A)	GCGGCCGCGUACAUCCAGCUGAUGAGUCCCAAAUAGGAC**A**AAACGGCGAAAGCCGUCCUGGAUUCCAGGCGGCCGC

HHR(m)	GCGGCCGCGUACAUCCAGCUGAUGAGUCCCAAAUAGGACGA**G**ACGGCGAAAGCCGUCCUGGAUUCCAGGCGGCCGC
OzS4	GCGGCCGCGUACAUCCAGCUGAUGAGUCCCAAAUAGGACGAAA**GUUCAGCAGCGAGC**UCCUGGAUUCCAGGCGGCCGC
OzS4 (G12A)	GCGGCCGCGUACAUCCAGCUGAUGAGUCCCAAAUAGGAC**A**AAA**GUUCAGCAGCGAGC**UCCUGGAUUCCAGGCGGCCGC
OzS4M21	GCGGCCGCGUACAUCCAGCUGAUGAGUCCCAAAUAGGACGAAA**GUUCAGCUGCGAGC**UCCUGGAUUCCAGGCGGCCGC
OzS4M21 (G12A)	GCGGCCGCGUACAUCCAGCUGAUGAGUCCCAAAUAGGAC**A**AAA**GUUCAGCUGCGAGC**UCCUGGAUUCCAGGCGGCCGC
OzS5	GCGGCCGCGUACAUCCAGCUGAUGAGUCCCAAAUAGGACGAAA**GGUUCAGCAGCGAGCC**UCCUGGAUUCCAGGCGGCCGC
OzS5 (G12A)	GCGGCCGCGUACAUCCAGCUGAUGAGUCCCAAAUAGGAC**A**AAA**GGUUCAGCAGCGAGCC**UCCUGGAUUCCAGGCGGCCGC
OzS5M21	GCGGCCGCGUACAUCCAGCUGAUGAGUCCCAAAUAGGACGAAA**GGUUCAGCUGCGAGCC**UCCUGGAUUCCAGGCGGCCGC
OzS5M21 (G12A)	GCGGCCGCGUACAUCCAGCUGAUGAGUCCCAAAUAGGAC**A**AAA**GGUUCAGCUGCGAGCC**UCCUGGAUUCCAGGCGGCCGC

Table S4. RNA molecules used in the RiboGreen interaction assay. In bold: motif 3 sequence; in bold and gray: mutation yielding inactive aptamer (M21); in bold and red: mutation yielding inactive HHR.

Name	Sequence 5´→ 3´
58	GGGAGGACGAUGCGGUG**UUCAGCAGCGAG**CACGUCGCGUCUACCCCGGUGAUACUCAGACGACUCGCUGAGGAUCCGAGA
58M21	GGGAGGACGAUGCGGUG**UUCAGCUGCGAG**CACGUCGCGUCUACCCCGGUGAUACUCAGACGACUCGCUGAGGAUCCGAGA
HHR(m)	GCGGCCGCGUACAUCCAGCUGAUGAGUCCCAAAUAGGACGA**G**ACGGCGAAAGCCGUCCUGGAUUCCAGGCGGCCGC
OzS4	GCGGCCGCGUACAUCCAGCUGAUGAGUCCCAAAUAGGAC**A**AAA**GUUCAGCAGCGAGC**UCCUGGAUUCCAGGCGGCCGC
OzS4M21	GCGGCCGCGUACAUCCAGCUGAUGAGUCCCAAAUAGGAC**A**AAA**GUUCAGCUGCGAGC**UCCUGGAUUCCAGGCGGCCGC
OzS5	GCGGCCGCGUACAUCCAGCUGAUGAGUCCCAAAUAGGAC**A**AAA**GGUUCAGCAGCGAG**CCUCCUGGAUUCCAGGCGGCCGC
OzS5M21	GCGGCCGCGUACAUCCAGCUGAUGAGUCCCAAAUAGGAC**A**AAA**GGUUCAGCUGCGAGCC**UCCUGGAUUCCAGGCGGCCGC

Table S5. List of shRNA constructs. In bold: motif 3 sequence; in orange: Hinge region nucleotides.

Construct	Sequence 5´→ 3´
SHA	GUAGCUUAUCAGACUGAUGUUGA**CGGUACAGCAGCGAUGCCG**CAACACCAGUCGAUGGGCUGUUU
m3_1	GCAAGCUGACCCUGAAGUUCAUAGAG**GGUUCAGCAGCGAGCC**AAUGAACUUCAGGGUCAGCUUGCUU
m3_2	GCAAGCUGACCCUGAAGUUCAUU**GGUUCAGCAGCGAGCC**AUGAACUUCAGGGUCAGCUUGCUU
m3_3	GCAAGCUGACCCUGAAGUUCAUUG**GGUUCAGCAGCGAGCC**CAUGAACUUCAGGGUCAGCUUGCUU

Table S6. List of oligonucleotides for the construction of sgRNA 58 and sgRNA 58 sequence. In bold: motif 3 sequence.

Construct	Sequence 5´→ 3´
NM60_f1	GGGGGAATTCTAATACGACTCACTATAGGGAAAGGTCGAGAAACTGCAAAGTTTTAGAGCTAGGCCAGGCCGCCGCTTCAGCAGCGAGGCGGCGGCCCTGCAGGGCCTAGCAAGTTAAAATAAGGCTAG
NM60_f2	AAAAAGCACCGACTCGGTGCCACTTGGCCCTGCAGGCGCGGCGGCTCGCTGCTGAACCGCCGCGCTGGCCAAGTTGATAACGGACTAGCCTTATTTTAACTTGCTAGGCCCTGCAG
sgRNA 58	GGGAAAGGUCGAGAAACUGCAAAGCUAGGCCAGGCCGCCGC**UUCAGCAGCGA**GGCGGCGGCCCUGCAGGGCCUAGCAAGUUAAAAUAAGGCUAGUCCGUUAUCAACUUGGCCAGCGCGGCGG**UUCAGCAGCGA**ACCGCCGCGCCUGCAGGGCCAAGUGGCACCGAGUCGGUGCUUUUU
